# The influence of hyperthermia on intracranial pressure, cerebral oximetry and cerebral metabolism in traumatic brain injury

**DOI:** 10.1080/03009734.2017.1319440

**Published:** 2017-05-02

**Authors:** Lena Nyholm, Tim Howells, Anders Lewén, Lars Hillered, Per Enblad

**Affiliations:** Department of Neuroscience/Neurosurgery, Uppsala University Hospital, Uppsala, Sweden

**Keywords:** Cerebral oximetry, hyperthermia, ICP, microdialysis, traumatic brain injury

## Abstract

**Background:**

Hyperthermia is a common secondary insult in traumatic brain injury (TBI). The aim was to evaluate the relationship between hyperthermia and intracranial pressure (ICP), and if intracranial compliance and cerebral blood flow (CBF) pressure autoregulation affected that relationship. The relationships between hyperthermia and cerebral oximetry (B_ti_pO_2_) and cerebral metabolism were also studied.

**Methods:**

A computerized multimodality monitoring system was used for data collection at the neurointensive care unit. Demographic and monitoring data (temperature, ICP, blood pressure, microdialysis, B_ti_pO_2_) were analyzed from 87 consecutive TBI patients. ICP amplitude was used as measure of compliance, and CBF pressure autoregulation status was calculated using collected blood pressure and ICP values. Mixed models and comparison between groups were used.

**Results:**

The influence of hyperthermia on intracranial dynamics (ICP, brain energy metabolism, and B_ti_pO_2_) was small, but individual differences were seen. Linear mixed models showed that hyperthermia raises ICP slightly more when temperature increases in the groups with low compliance and impaired CBF pressure autoregulation. There was also a tendency (not statistically significant) for increased B_ti_pO_2_, and for increased pyruvate and lactate, with higher temperature, while the lactate/pyruvate ratio and glucose were stable.

**Conclusions:**

The major finding was that the effects of hyperthermia on intracranial dynamics (ICP, brain energy metabolism, and B_ti_pO_2_) were not extensive in general, but there were exceptional cases. Hyperthermia treatment has many side effects, so it is desirable to identify cases in which hyperthermia is dangerous. Information from multimodality monitoring may be used to guide treatment in individual patients.

## Introduction

Hyperthermia is defined with different temperature thresholds in the literature (>38.5 °C, >38.3 °C, >30.0 °C, and >37.5 °C) and is reported to occur in 15%–80% of patients with traumatic brain injury (TBI) (1–6). Hyperthermia is considered to be a secondary systemic insult to the injured brain and is therefore treated aggressively (7,8). If hyperthermia occurs the first week after injury it is associated with long-term poor outcome (9–11). The amount of early hyperthermia is associated with the severity of TBI (3,9). Limited knowledge exists regarding mechanisms behind the negative effects of hyperthermia in TBI patients. There are inconsistent results regarding the negative effects of hyperthermia on intracranial pressure (ICP), cerebral perfusion pressure (CPP) (4,12–15), and brain tissue oxygenation (B_ti_pO_2_) (16,17). Clearly there is a need for better understanding of the effects of hyperthermia on intracranial dynamics in TBI patients. The main aim of this study was to evaluate the relationship between hyperthermia and ICP and the influence of intracranial compliance and cerebral blood flow (CBF) pressure autoregulation. Secondary aims were also to study the relationships between hyperthermia and cerebral oxygenation and cerebral metabolism.

## Materials and methods

### Patients

All patients with TBI treated at the neurointensive care (NIC) unit, Uppsala University Hospital, from January 2008 to December 2010 were eligible if they were mechanically ventilated and had simultaneous ICP and temperature monitoring. Clinical data were gathered from the Uppsala TBI register (18). The study included 87 patients (17 women and 70 men) aged 15–80 years (median 41.0). On admission to the NIC unit the median Glasgow coma scale–motor score (GCS-M) was 5. Primary dominating findings on the initial CT scan were contusions (47%), acute subdural hematoma (15%), mixed injuries (14%), diffuse axonal injury (12%), epidural hematoma (6%), traumatic subarachnoid hemorrhage (3%), and other (3%). The patients stayed at the NIC unit for 4–62 days (median 15).

### Management of TBI at the NIC unit

Patients were treated according to a standardized escalated management protocol (19). The goals were to keep ICP <20 mmHg and CPP ≥60 mmHg, and to avoid other secondary insults (19). All patients who were not responding to commands (GCS-M ≤5) should be intubated and have ICP monitoring. The artificially ventilated patients received propofol and morphine chloride. The patients’ heads were elevated (∼30°) to facilitate venous outflow and to avoid ventilator-associated pneumonia. Significant mass lesions were evacuated. If ICP remained elevated despite this basal treatment, cerebrospinal fluid drainage, thiopental coma treatment, and external decompressive craniectomy were used in an escalated order.

According to the standardized procedures at the NIC unit, the core temperature should be kept below 38 °C. Strict hygiene routine should be followed to avoid infections. Furthermore, patients should be properly sedated and pain relieved to avoid stress. No warm clothes or bed linen should be used. The recommended treatment of hyperthermia was acetaminophen, then cooling blankets, and finally infusions of thorazine.

### The Odin monitoring system

All patients at the NIC unit are connected to the Odin monitoring system (20) developed by Tim Howells and colleagues. The system collects and stores high-resolution (up to 200 Hz) monitoring data every minute with exact time stamps. It includes functions for analysis of monitoring data in relation to clinical data, statistical analysis, and presentation of the results of the data analysis.

### Monitoring data

High-resolution monitoring data from start of monitoring (in practice when all required catheters were in place) and until either temperature or ICP monitoring was ended were used up to a maximum of 10 days. Median individual monitoring time was 5.3 days (range 0.18–9.89). Median time between the trauma and start of monitoring was 13 hours (range 2–89). The definition of good monitoring time (GMT) is the time left when all gaps (e.g. during surgery and radiology) in monitoring data are removed. When ICP, compliance, or pressure reactivity index (PRx) was calculated, all monitoring data after the first opening of the ventricular drainage system, decompressive craniectomy, and/or thiopental coma treatment, respectively, were excluded. The reason for this was that it is unlikely that fever increases ICP after decompressive craniectomy or when the ventricular drainage system is opened due to increased compliance, and thiopental both increases compliance and reduces temperature.

All temperatures below 36.0 °C (<4% of GMT) were excluded to reduce the risk of including artifacts, for example from flushing the catheter with NaCl. If ICP was >50 mmHg (<1% of GMT) the data were excluded in order to avoid bias in case of withdrawal of treatment.

### Temperature

Temperature was monitored with a probe in the urinary catheter (Mon-a-Therm™ Foley catheter with temperature sensor 400TM, Covidien, Germany). This catheter measures temperature in a range of 25–45 °C, with a tolerance of ±0.1 °C within 150 seconds, according to the specifications by the manufacturer.

### Intracranial pressure and intracranial compliance

A ventricular drainage catheter system was used for ICP measurements if possible (Smiths Medical, Germany). The zero calibration level of the ICP transducer was adjusted to 2 cm below the highest point of the skull (21). In cases with compressed ventricular system a parenchymal probe was used instead (Codman ICP express®, Johnson & Johnson, USA or Neurovent-PTO, Raumedic AG, Germany) (22,23). Fifteen patients had ventricular drainage catheters, 51 patients had parenchymatous ICP transducers (35 Codman and 16 Neurovent-PTO), and 21 had both (ICP measured from the ventricular drainage catheter).

Intracranial compliance was estimated by using the amplitude of the ICP curve (24). Poor compliance was defined as amplitude >6 mmHg (24).

### Cerebral blood flow pressure autoregulation

PRx (25) is based on the correlation of ICP and mean arterial pressure (MAP), so that when ICP is highly correlated with MAP, PRx approaches a maximum value of one. Usually PRx is computed from the ICP and MAP waveforms by taking the average values of a series of 5–10-second segments over a total period of 3–5 minutes. Correlation of the two series is computed to produce the index. Sometimes the waveforms are preprocessed using a bandpass filter to remove irrelevant low- and high-frequency variation (24,26). In the current study we have used the methodology described by Howells et al. (27), which uses a filter with a bandpass of 0.018–0.07 Hz. Thus, we limit the correlation analysis to oscillations of MAP and ICP with periods of 15 to 55 seconds in duration.

A PRx threshold of >0.3 was used to distinguish patients with clearly disturbed pressure autoregulation. The applied threshold was based on the study by Sorrentino et. al. showing fatal outcome when PRx >0.25 (25).

### Cerebral oximetry

A Neurovent-PTO probe was used in 16 patients (28–30). Every patient had at least 13.3 hours’ monitoring time of B_ti_pO_2_. The probe was routinely placed via a burr hole in the right frontal lobe. If a hemicraniectomy or evacuation of mass lesion was done on the left side the probe was placed in the left hemisphere.

### Cerebral energy metabolism

Intracerebral MD was used in 28 patients for neurochemical monitoring. The MD catheter (71 High Cut-Off Brain Microdialysis Catheter®, M Dialysis AB, Sweden) was placed close to the pressure device in normal brain tissue. The catheter was connected to a CMA 106 or 107 microinfusion pump® (M Dialysis AB). The perfusion fluid rate was 0.3 μL/min, and the samples were collected every hour. The 28 patients accumulated 2555 samples in total (median 91, range 6–272). Glucose, lactate, pyruvate, and urea were analyzed using a bedside analyzer CMA 600® (CMA Microdialysis, Sweden). The lactate/pyruvate (L/P) ratio was calculated. Quality control measurements, using control samples for the CMA 600 MD Analyzer, were run daily. Total imprecision for all analyses had a coefficient of variation (CV) of <10%. Probe performance was validated by monitoring interstitial fluid (ISF) urea (31).

When relating MD values to the mean temperature for the previous hour, the MD time was adjusted to account for a lag of 17 minutes due to MD catheter dead space.

The MD pattern was classified in four categories for each patient, based on published normal and critical MD levels (32–35): ischemia (L/P ratio >40 and pyruvate <50 μmol/L), energy metabolic crisis (L/P ratio >25 and pyruvate 50–120 μmol/L), and hyperglycolysis (L/P ratio <40, lactate >4, and pyruvate >120 μmol/L). The percentage of samples in each of the MD categories was calculated for each patient. Furthermore, the MD samples were divided into high (38–42 °C) and low (36–37.9 °C) temperature.

### Statistical methods

*Between-group comparison*. For comparing the proportion of GMT with ICP >20 mmHg in temperature groups (<39 °C/≥39°) and for comparing MD values within MD groups, the Wilcoxon signed rank test was used. For comparing age and GCS-M, the Mann–Whitney test was used. Differences were considered to be statistically significant when *p* < 0.05.

*Mixed models*. In order further to explore the relationship between variables of interest, linear mixed models were evaluated. In all models hyperthermia was included as a fixed factor and patient as a random factor. In order to determine the effect of hyperthermia depending on different confounding factors, the first model for each factor included the interaction between hyperthermia and the factor of interest.

In case of a non-significant interaction, the interaction term was excluded from the model, and the final model contained the main effect for hyperthermia and the factor of interest. For all models the predicted values and 95% confidence intervals from the final model are presented in figures.

### Ethics

The patients were unconscious when they participated in the studies. Informed consent was obtained from their relatives. The Uppsala Regional Ethical Review Board granted permission (Dnr 2004:M-140).

## Results

### The amount of hyperthermia

Sixty-eight patients (78%) had hyperthermia (>38 °C) more than one hour during the first 10 days after start of monitoring. Median proportion of GMT was 20% for hyperthermia >38 °C but ≤39 °C and 2% for hyperthermia >39 °C but ≤42 °C.

### Hyperthermia and intracranial pressure

**Figure 1.**
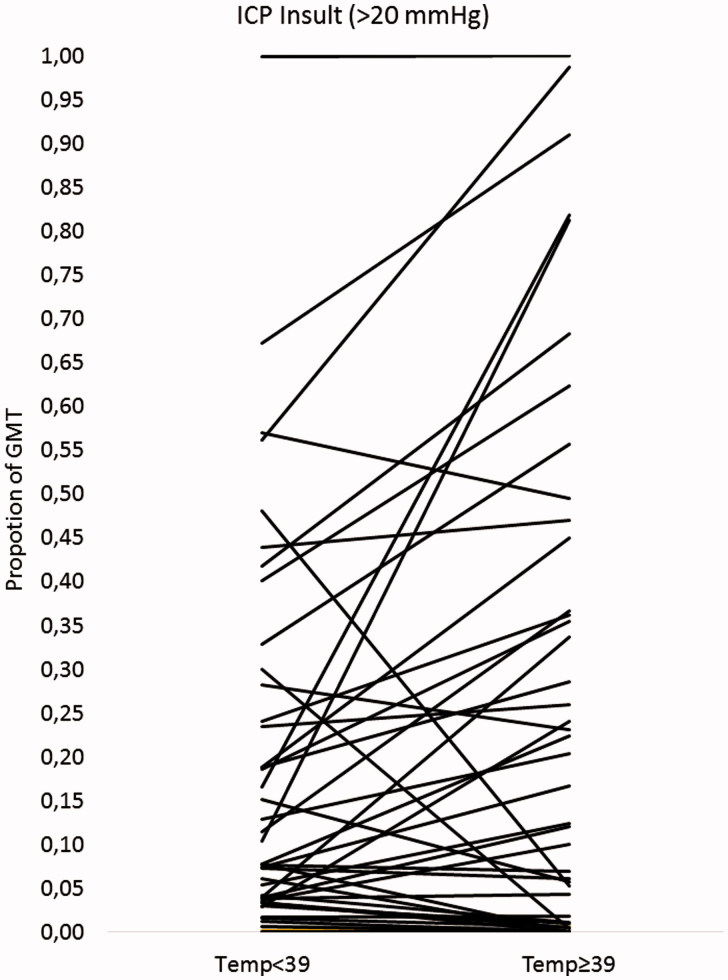
The occurrence of intracranial pressure (ICP) > 20 mmHg (in proportion of good monitoring time [GMT]) compared between periods in the highest temperature range (temperature ≥39 °C) and periods in the lower ranges (temperature <39 °C) for individual patients (25 patients with time in both temperature ranges). Each line with dots represents one patient.

When the occurrence of ICP insults (≥20 mmHg) was compared between periods with temperature ≥39 °C and periods with temperature <39 °C in individual patients (25 patients with time in both temperature ranges), there was a significantly (*p* = 0.025) larger proportion of GMT with ICP ≥20 mmHg in the higher temperature ranges (Figure 1). The median increase (difference in proportion of GMT with ICP ≥20 mmHg) was 1%, and 5 (20%) of the patients had increases over 25%.

### Hyperthermia, intracranial compliance, and ICP

**Figure 2.**
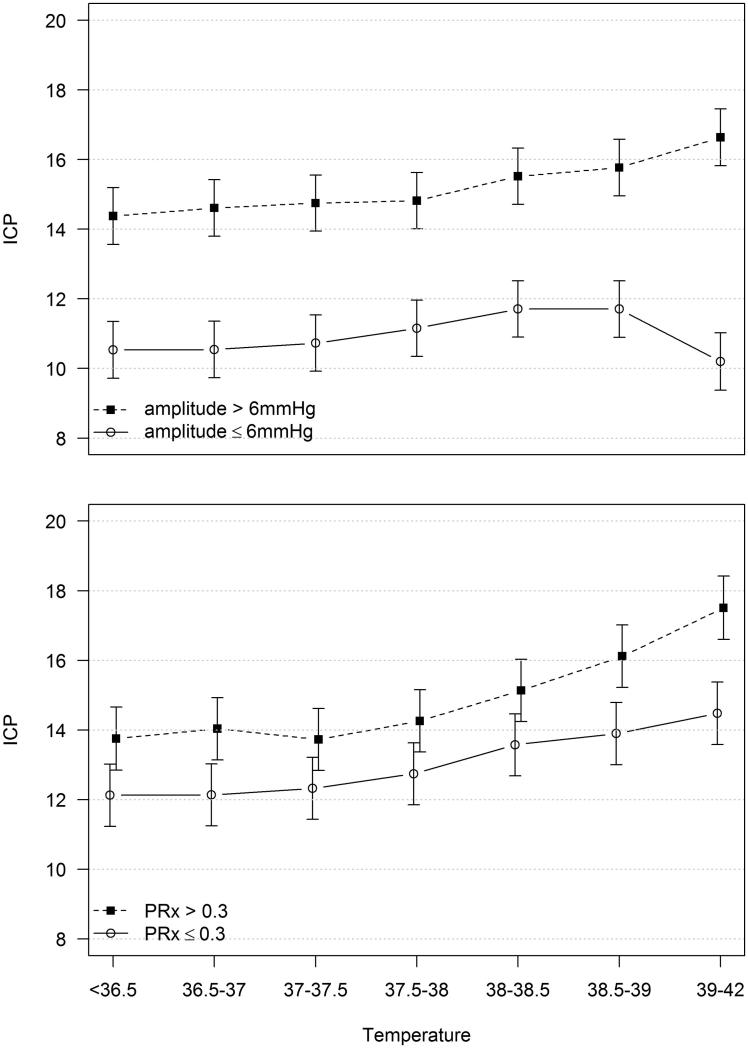
Predicted values (and 95% CI) from a mixed model including intracranial pressure (ICP) (mmHg) as dependent variable. Upper panel, temperature (°C), compliance (amplitude > or ≤6 mmHg), and the interaction between temperature and compliance as independent variables. Lower panel, temperature (°C), pressure autoregulation (PRx > or ≤0.3) and the interaction between temperature and pressure autoregulation as independent variables.

The estimates of the mixed model including ICP, temperature, and compliance are shown in Figure 2 with 95% confidence intervals. Seven temperature ranges are included, with the highest being 39–42 °C. In the group with poor compliance, ICP increased approximately 2 mmHg and was significantly elevated in the highest temperature range compared to the four lowest ranges. In the group with good compliance, the changes in ICP with increased temperature were not statistically significant.

### Hyperthermia, pressure autoregulation, and intracranial pressure

The mixed model showed that ICP was higher overall in the group with impaired pressure autoregulation (PRx >0.3), and that it also increased more with increasing temperature (Figure 2). In the impaired group, mean ICP increased approximately 4 mmHg. The increase was statistically significant when comparing the highest temperature range to any of the five lowest ranges, or comparing the second highest range with the four lowest. In the group with preserved pressure autoregulation, the increase in ICP was approximately 3 mmHg, and the increase was significant only when comparing the highest range to the three lowest ranges (Figure 2).

### Hyperthermia and cerebral oximetry

**Figure 3.**
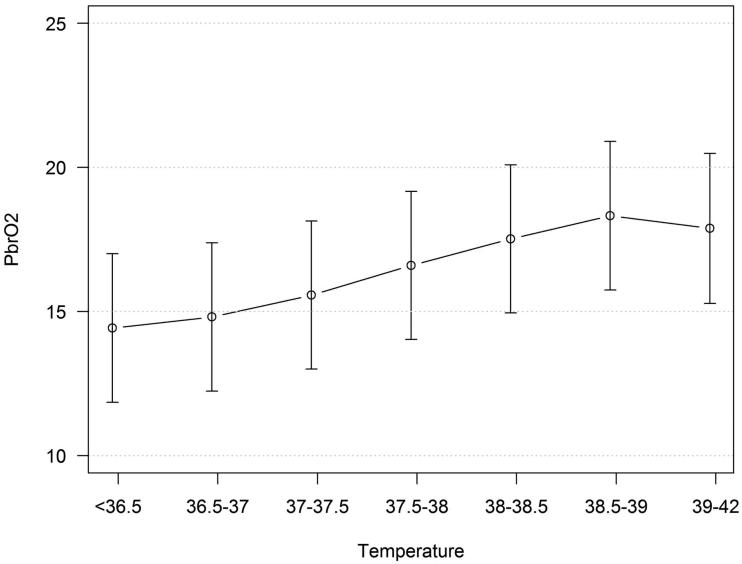
Predicted values (and 95% CI) from a mixed model including B_ti_pO_2_ (mmHg) as dependent variable and temperature (°C) as independent variable.

A mixed model including temperature and B_ti_pO_2_ (dependent variable) showed that B_ti_pO_2_ increased when temperature increased (Figure 3), although the changes were not statistically significant. The relationship between B_ti_pO_2_ and temperature was not influenced by ICP.

### Hyperthermia and cerebral energy metabolism

**Figure 4.**
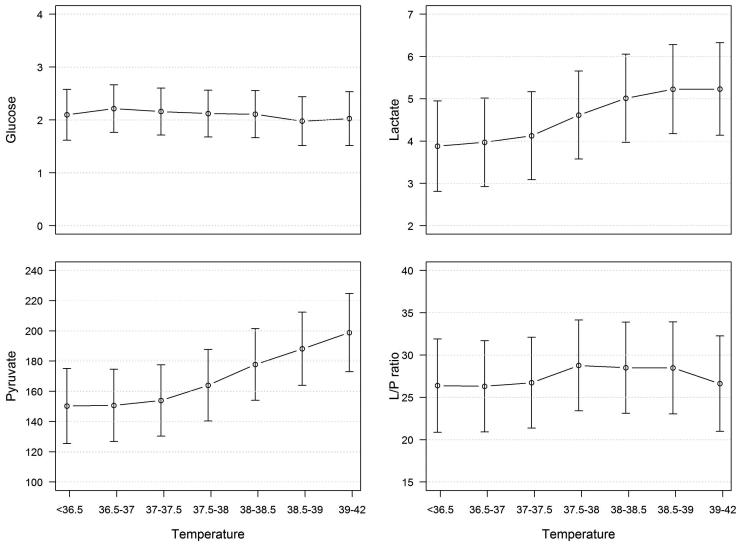
Predicted values (and 95% CI) from mixed models including energy metabolic microdialysis substances (glucose, lactate, pyruvate, and L/P ratio) as dependent variables and temperature (°C) as independent variable. L/P ratio = lactate/pyruvate ratio.

A mixed model (compensating for ICP) including temperature and all energy metabolic MD substances (dependent variables) was made. Temperature did not influence glucose or L/P ratio (Figure 4). Lactate and pyruvate increased when temperature increased (Figure 4), although the changes were not statistically significant.

The hourly MD sample for each patient (*n* = 28) was categorized into the metabolic patterns previously described. Ischemia was never seen, energy metabolic crisis was seen in 11% (271/2555) of the samples, hyperglycolysis in 34% (862/2555), and normal energy metabolism in 55% (1422/2555). The relative occurrence (proportions of samples) in individual patients of each MD category by temperature is shown in Table 1. There were no significant differences in the occurrence of MD categories depending on whether the patients had temperature 36–38 °C or 38–42 °C (temperatures <36 °C and >42 °C excluded to avoid inclusion of artifacts) when the MD sample was collected (Table 1).

**Table 1.** The median relative occurrence (proportions of samples) for all individual patients in each microdialysis category (ischemia, metabolic crisis, hyperglycolysis, normal) by temperature. There were 1894 samples without hyperthermia in 28 patients and 661 with hyperthermia in 22 of the 28 patients.

	Temperature^a^		
	36 °C–37.9 °C	38 °C–42 °C	
Microdialysis categories^b^	Median occurrence^c^ % (IQR)	Median occurrence^c^ % (IQR)	*P* value
Ischemia	0 (0)	0 (0)	–
Metabolic crisis	0 (1)	0 (21)	0.42
Hyperglycolysis	34 (73)	26 (92)	0.65
Normal	55 (87)	44 (78)	0.63

a Body temperature when microdialysis sample collected.

b Ischemia: L/P ratio >40 and pyruvate <50; Metabolic crisis: L/P ratio >25 and pyruvate 50–120; Hyperglycolysis: L/P ratio <40, lactate >4, and pyruvate >120.

c The relative occurrence of the microdialysis categories was calculated for each patient, and then the median relative occurrence for all patients was calculated.

## Discussion

Occurrence of hyperthermia among the patients in this study seems to be at the same level as in most other NIC units (1–6,36). The effects of hyperthermia on intracranial dynamics (ICP, brain energy metabolism, and B_ti_pO_2_) were in general small, but individual differences were seen.

When studying the effects of hyperthermia on ICP in TBI patients it is important to take into account the complexity of the intracranial dynamics (37) including factors such as the pressure–volume relationship and intracranial compliance, and the pressure autoregulation of CBF. Our working hypothesis was that hyperthermia may lead to elevated intracranial pressure. Hyperthermia may increase cerebral metabolism (38,39), i.e. increased metabolic demand. The coupling between metabolism and CBF will increase CBF as well as the cerebral blood volume (CBV), which could cause an elevation of ICP. Furthermore, hyperthermia may increase the stress level of the patient, resulting in increased cardiac output and blood pressure, which may increase CBF and CBV, especially under conditions of impaired pressure autoregulation (see below) (6,40). Elevation of CBV may cause elevated ICP, and this risk could be greatest for patients with poor intracranial compliance.

To address the question whether hyperthermia caused clinically significant increases in ICP we compared the occurrence of ICP insult (≥20 mmHg) during periods of high temperature (≥39 °C) with periods of low temperature (<39 °C). The result shows that high temperature was associated with increased risk of ICP insults. For most patients, however, the increased risk was negligible, since the median increase (difference in proportion of GMT with ICP >20 mmHg) was only 1%. On the other hand, the increased secondary insult was of potential clinical significance in several cases (20% of the patients had increases over 25%). It is obvious that the ICP response to hyperthermia differs between the patients.

We assumed that hyperthermia would increase CBF (and thereby CBV) both through the metabolic regulation of CBF and the increased level of stress. Our idea was that the increased CBV only would result in an elevation of ICP in patients with decreased compliance. The mixed model showed that ICP increased by increasing temperature in both compliance groups, but the increase was only significant in the group with poor intracranial compliance. This finding indicates that the ICP reaction to hyperthermia is influenced by compliance, although the average effect is small from a clinical perspective. Further studies are required to elucidate to which extent compliance determines the ICP response of hyperthermia.

We hypothesized that an increase of blood pressure due to an increased stress level caused by hyperthermia would increase CBV (and ICP) more in patients with impaired autoregulation. ICP increased significantly by increasing temperature in both groups, and the increases were greater in the group with impaired pressure autoregulation.

It is reasonable to believe that hyperthermia should not influence B_ti_pO_2_ due to the flow energy metabolism coupling, especially if the treatment is adequate and sufficient substrate is delivered. Theoretically, increased metabolism by hyperthermia could result in a raised oxygen extraction with decreased B_ti_pO_2_ if CBF is not increased enough by the metabolic regulation. On the other hand, an increased level of stress, caused by hyperthermia, may raise the blood pressure which could result in increased CBF and increased supply of O_2_ to brain tissue reflected by increased B_ti_pO_2_.

The overall picture found in the analysis was that B_ti_pO_2_ increased with increasing temperature. This result is the same as in one other previous study (17). The increase of B_ti_pO_2_ is probably an effect of both preserved coupling between metabolism and flow and increased CBF due to stress, especially if pressure autoregulation is impaired. The results may also have been influenced by the Hb–O_2_ dissociation temperature dependency (41).

Hyperthermia may probably be both the result and the cause of an increased energy metabolism. Under physiological conditions with increased energy demand, the coupling between metabolism and CBF results in an increased CBF and substrate delivery. If there is an uncoupling between energy metabolism and CBF, CBF may become either too high (hyperperfusion) or too low (ischemia or energy metabolic crisis) in relation to the energy metabolism. Furthermore, hyperglycolysis may also occur.

In this study the MD did not show ischemia, but energy metabolic patterns indicating normal metabolism, non-ischemic energy metabolic crisis, and hyperglycolysis were seen. Normal energy metabolism and hyperglycolysis were most common, and non-ischemic energy metabolic crisis was rare. The mixed linear model showed a slight increase of both pyruvate and lactate by increasing temperature (statistically not significant), while the L/P ratio and glucose were relatively stable, which may indicate that the occurrence of hyperglycolysis is higher during hyperthermia. It is possible that the results would have been different if the MD probes had been placed in disturbed brain tissue, i.e. ‘tissue at risk’.

The main results of this study indicate that the increase of ICP during hyperthermia is small in general, but there appears to be individual differences where some patients may develop high ICP. The different individual ICP reactions to hyperthermia appear to be influenced by intracranial compliance and pressure autoregulation to some extent. Another important observation was that ischemic patterns in the MD samples were never seen. These findings are novel knowledge, but it must be realized that the results may possibly have been different if hyperthermia and ICP had been untreated. It is also possible that hyperthermia treatment in itself may have influence, e.g. in situations of shivering when cooling blankets are used or in hypotensive events associated with paracetamol infusion. Furthermore different causes of hyperthermia may also influence ICP differently as well as the overall injury severity. Other methodological problems, which are difficult to overcome, are that ICP and temperature have different temporal characteristics (ICP can change quickly, while temperature changes slowly) and that ICP may increase for many different reasons in addition to hyperthermia. These problems may also partly explain inconsistent results between different studies (4,12–15).

Finally, it is of great interest to discuss if hyperthermia always should be treated. The effects of hyperthermia on ICP, brain energy metabolism, and B_ti_pO_2_ differed between patients, indicating that hyperthermia may not always be dangerous. It is also difficult to treat hyperthermia, and there are side effects of the treatment, e.g. shivering (42,43). Patients treated with a surface-cooling device have to be deeply sedated, and this increases the risk for low blood pressure, while the sedation also obstructs neurological wake-up tests. Acetaminophen is often used to treat hyperthermia but can be associated with hepatic toxicity. Treating hyperthermia may also hide the symptoms of an infection and therefore delay treatment of infections (43). One way to identify which patient would benefit from reducing hyperthermia is to use multimodality monitoring devices. If a TBI patient with hyperthermia has problems with e.g. ICP, B_ti_pO_2_, or cerebral energy metabolism, hyperthermia should be treated. Further studies are needed on this topic to obtain better guidelines on when hyperthermia should be treated in order to get a more individualized approach.

## Conclusion

The major finding in this study was that the effects of hyperthermia on intracranial dynamics (ICP, brain energy metabolism, and B_ti_pO_2_) were not extensive in general, but there was an individual variation. Since the treatment of hyperthermia has many side effects, it is desirable to gain more knowledge about which hyperthermia episodes are dangerous and should be treated. Information from multimodality monitoring devices may be used to guide treatment.
